# Superficial Calcified Plates Associated to Plaque Erosions in Acute Coronary Syndromes

**DOI:** 10.3390/life13081732

**Published:** 2023-08-11

**Authors:** Horea-Laurentiu Onea, Mihail Spinu, Calin Homorodean, Mihai Claudiu Ober, Maria Olinic, Florin-Leontin Lazar, Alexandru Achim, Dan Alexandru Tataru, Dan Mircea Olinic

**Affiliations:** 1 Medical Clinic Number 1, “Iuliu Hatieganu” University of Medicine and Pharmacy, 400012 Cluj-Napoca, Romania; onea.lau@gmail.com (H.-L.O.); chomorodean@yahoo.com (C.H.); maria_olinic@yahoo.com (M.O.); lazar.leontin@yahoo.com (F.-L.L.); tataru.cardio@gmail.com (D.A.T.); danolinic@gmail.com (D.M.O.); 2 Department of Interventional Cardiology, Cluj County Emergency Hospital, 400006 Cluj-Napoca, Romania; mihai_ober@yahoo.com; 3 Cardiology Department, Kantonsspital Baselland, 4410 Liestal, Switzerland; dr.alex.achim@gmail.com

**Keywords:** atherosclerotic coronary disease, acute coronary syndromes, optical coherence tomography, culprit lesions, plaque erosion, superficial calcified plates

## Abstract

This study investigates the clinical relevance and therapeutic implications of the OCT identification of intracoronary superficial calcified plates (SCPs) in acute coronary syndromes (ACSs). In 70 consecutive ACS patients (pts), we studied the three main underlying ACS mechanisms: plaque erosion (PE), plaque rupture and eruptive calcified nodule (CN). The PE lesions, occurring on an intact fibrous cap overlying a heterogeneous substrate, were identified in 12/70 pts (17.1%). PE on superficial calcified plates (PE-SCP) represented 58.3% of the PE lesions (7/12 pts) and had a 10% overall incidence in the culprit lesions (7/70 pts). PE-SCP lesions occurred mostly on the left anterior descending artery, correlated with white thrombi (85.7%) and had a proximal intraplaque site (71.4%). PE-SCP lesions were treated conservatively, as nonsignificant lesions, in 4/7 pts. Our study emphasizes that the coronary calcium-related ACS risk is not only associated with the spotty calcifications or CN but also with the PE-SCP lesions.

## 1. Introduction

Acute coronary syndromes (ACS) encompass a spectrum of clinical presentations and pathophysiological mechanisms all leading to various degrees of impaired blood flow to the myocardium [1]. Intravascular imaging, mainly through optical coherence tomography (OCT), represents a useful diagnostic tool in the setting of ACS, providing good analytical capacity for culprit plaque morphology and can even detect non-atherosclerotic lesions. Equally as important, this technique can help guide percutaneous coronary interventions (PCI) from procedure indication, choice of adequate treatment strategy and advanced stent optimization and complication management [2,3,4,5,6,7,8].

Three main mechanisms of plaque destabilization have been contemporarily described [9] and include plaque erosion (PE), plaque rupture and eruptive calcified nodule (CN). By means of OCT, PE is defined as the presence of a thrombus overlying an intact fibrous cap and has been found in equal measure on both fibrous and lipid-rich plaques [10]. Calcium-related risk was mainly studied in association with CN, which is an intraluminal protruding calcium mass found in up to 8% of ST-segment elevation myocardial infarction (STEMI) cases [11].

Our study investigates the coronary risk pertaining to another calcium-related lesion, the superficial calcified plate (SCP), which is defined as sheet-like superficial calcium without erupted nodules or protruding mass into the lumen [12]. Using OCT imaging, we analyzed the clinical relevance and therapeutic implications of SCP identification in relation to PE in the culprit lesions of patients with ACS.

## 2. Materials and Methods

### 2.1. Study Population

This was an observational retrospective study conducted at a tertiary-care center in Romania (Cluj County Emergency Hospital, Department of Interventional Cardiology) between January 2012 and May 2021. Consecutive ACS patients ≥18 years old with an indication of coronary angiography (CA) who underwent OCT imaging of the culprit lesion after angiography were included.

STEMI, non-ST-segment elevation myocardial infarction (NSTEMI) and unstable angina pectoris (UAP) were defined in accordance with the relevant guidelines [13].

Patients with poor OCT image quality, in-stent complication, non-atherosclerotic coronary lesions or unidentifiable culprit lesions were excluded.

Informed patient consent was obtained prior to the procedure. This study complies with the Declaration of Helsinki on human research.

All patients underwent clinical follow-up after the index procedure via telephone interviews, with a specific interest on all-cause mortality.

### 2.2. Coronary Angiography

CA was performed using the on-site existing angiograph: Siemens Artis Zee (Siemens Healthineers, Erlangen, Germany). Coronary stenosis severity was estimated both visually and by quantitative coronary analysis methods. The highest degree of stenosis was taken into consideration. After CA, the coronary stenoses were classified as significant (>70%), nonsignificant (<50%) or borderline (between 50% and 70%). Significant left main (LM) disease was defined in the presence of a >50% stenosis.

The culprit lesion was identified based on electrocardiographic changes, echocardiographic wall motion abnormalities and angiographic appearance. Multivessel disease was diagnosed in the presence of a significant stenosis in any of the non-culprit vessels or LM disease. Coronary flow was assessed according to the Thrombolysis in Myocardial Infarction score.

### 2.3. OCT Acquisition Technique and Image Analysis

OCT imaging was performed using the frequency-domain ILUMIEN^TM^ OPTIS^TM^ OCT system (St. Jude Medical, St. Paul, MN, USA). The optical catheter was advanced distally with respect to the lesion on a 0.019-inch guidewire. The manual injection of contrast media with subsequent blood displacement occurred simultaneously with automated probe pullback at a speed of 20 mm/s.

All OCT images were digitally archived in a dedicated database, the RoM1OCTRegistry. Two independent investigators blinded to the patients’ clinical and angiographic data analyzed the images, while a third reviewer resolved any dissonance between the former.

Intracoronary OCT images were analyzed in order to identify culprit lesions associated to the ACS. Once identified, the culprit lesion was studied in terms of plaque length, area stenosis and plaque morphology.

The total plaque length was measured as the distance from diseased-to-diseased segment. Quantitative plaque analysis was performed at 1 mm intervals. Proximal and distal references were identified as the sites with the largest lumen diameter/area proximal and distal to the plaque but within a 10 mm segment. The average between the proximal and distal references was calculated. Minimal lumen diameter (MLD) and minimal lumen area (MLA) were defined as the smallest diameter/area in the lesion segment. Percent diameter stenosis and area stenosis were determined as follows: [(average reference LD − MLD)/average reference LD] × 100 and [(average reference LA − MLA)/average reference LA] × 100 [14]. After OCT, coronary lesions were classified as significant or nonsignificant based on %area stenosis values: >70% or <50%, respectively. An MLA value of <6 mm^2^ was considered the cutoff for LM revascularization [15].

PE was identified by the presence of a thrombus or luminal irregularity without a clear rupture site [16]. Plaque rupture was diagnosed when there was fibrous cap discontinuity associated with a clear intraplaque cavity [16]. Thrombus was defined as an intraluminal mass that may or may not be attached to the luminal surface and was classified as red thrombus, defined by high attenuation and intense signal or white thrombus, characterized by low attenuation and homogeneous backscattering [16]. Healed plaque (HCP) was recognized by a single or multiple high-signal layers with different optical densities, near the luminal surface and with clear delimitation from the underlying tissue [16].

A lipid-rich plaque was characterized by lipid pools extending in at least two quadrants [16]. Thin-cap fibroatheroma was defined as a lipid-rich plaque surrounded by a thin (cutoff 65 µm) fibrous cap [16].

Calcium was identified by low attenuating and low signal regions with sharply delineated borders [16]. Eruptive CN was defined as an expulsion of small calcific nodules into the lumen. Calcified protrusion (CP) was defined as a protruding calcium mass into the lumen without eruptive nodules [12].

This study particularly investigated the SCP lesions, which are defined as non-protruding sheets of calcium encapsulated by a thin fibrous cap. We analyzed the prevalence of SCP within the culprit plaques and particularly within the subset of PE in relation with the clinical picture, intraplaque complication morphology and topography. The correlation with the subsequent treatment strategy was performed.

## 3. Results

In 70 ACS patients (17 STEMI, 19 NSTEMI and 34 UAP), the 3 main underlying ACS mechanisms were identified. PE was the culprit lesions’ morphological pattern in 12 patients (17.1%). PE occurring on SCP represented 58.3% of the total PE subgroup (7/12 patients) and had a 10% overall incidence in the culprit lesions (7/70 patients).

### 3.1. Clinical Data

Baseline characteristics of the seven PE-SCP patients are outlined in Table 1. The median age of the patients was 70 years, and four were female. With respect to the clinical diagnosis, three patients presented with NSTEMI and four presented with UAP. At admission, patient #3 associated an episode of ventricular tachycardia requiring medical cardioversion. Classical cardiovascular risk factors were present, ranging from 1 to 4 in each patient, whereas only two patients had chronic kidney disease. Two patients were free of vascular disease in any territory, while previous myocardial infarction and PCI was noted in three and four patients, respectively. The electrocardiogram showed clear ischemic changes (ST depression and negative T waves) in two patients. Culprit vessel-related regional wall motion abnormalities were observed in three patients. 

All patients were receiving antiplatelet and statin therapy before admission.

### 3.2. Coronary Angiography Data

Relevant imaging findings are shown in Table 2.

The seven patients with PE-SCP lesions on OCT had their culprit lesion on the left anterior descending artery (LAD) in four cases and LM in two cases, while patient #4 exhibited multiple concurring complication sites in both the LAD and LM. Multivessel disease status was present in 71.4% of cases. 

On CA, culprit lesions with PE-SCP aspect were significant only in one out of seven patients, while five other patients had borderline angiographic lesions and one patient presented a nonsignificant lesion.

**Figure 1:**
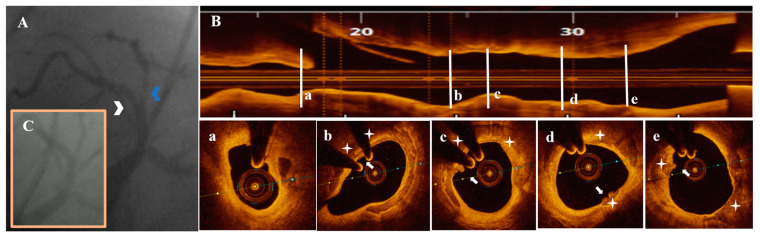
A 74-year-old patient presenting with UAP. (**A**) CA—borderline proximal LAD (white arrowhead) and significant proximal second diagonal (blue arrowhead) stenoses. (**B**) OCT—LAD plaque longitudinal view. (**a**) Significant (area stenosis = 73.9%) stenosis after the origin of the second diagonal. (**b**–**e**) SCP (white star) presenting PE with white thrombi (white arrow) in the proximal plaque segment. (**C**) CA—final result after PCI with 2.75 mm DES/LAD and plain old balloon angioplasty/second diagonal.

**Figure 2:**
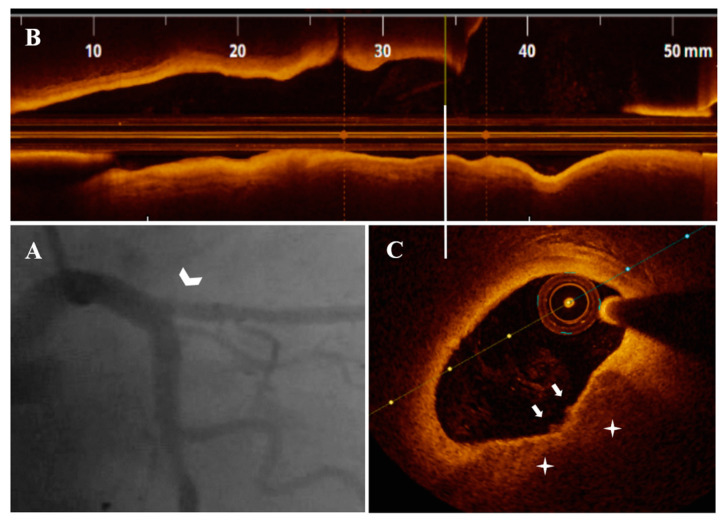
A 69-year-old patient presenting with UAP. (**A**) CA—nonsignificant, eccentric and hazy ostial LAD plaque (white arrowhead). (**B**) OCT—LAD plaque longitudinal view. (**C**) Nonsignificant ostial LAD plaque (area stenosis = 38%) with PE-SCP (white star) overlaid by white thrombi (white arrow).

**Figure 3:**
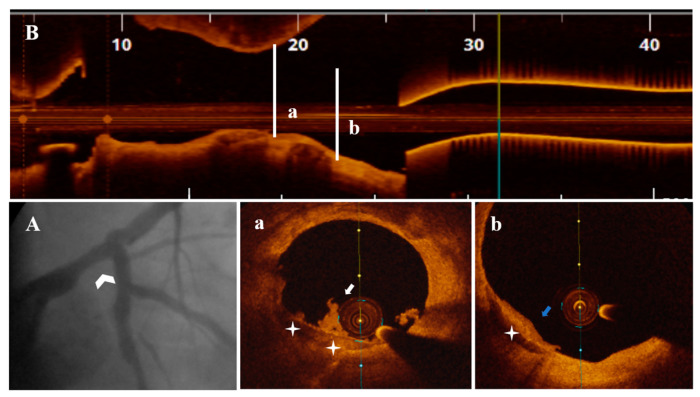
A 51-year-old patient presenting with NSTEMI. (**A**) CA—borderline mid-distal LM stenosis (white arrowhead). (**B**) OCT—LM plaque longitudinal view. (**a**,**b**) OCT—transversal view, nonsignificant mid-LM plaque (area stenosis = 42.9%) presenting PE with white thrombi (white arrow) on SCP (white star) extending proximally in interrelation with a healed plaque (blue arrow).

**Figure 4:**
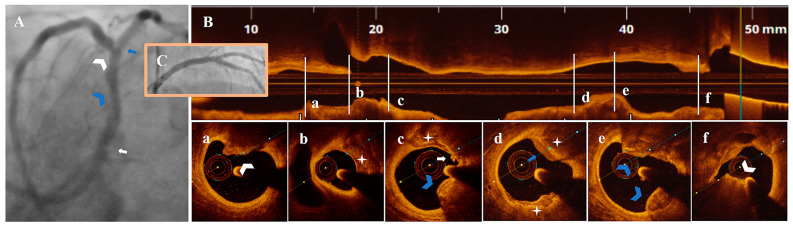
A 79-year-old patient presenting with NSTEMI. (**A**) CA—LM with significant proximal stenosis (white arrow), LAD with nonsignificant proximal (blue arrowhead) and severe mid-stenosis (white arrowhead), diagonal branch with borderline ostial stenosis (blue arrow). (**B**) OCT—LM-LAD plaque longitudinal view. (**a**–**c**) Severe mid-LAD lesion (AS = 82.6%) with eruptive CN (white arrowhead), calcified protrusion (blue arrowhead), SCP (white star) and PE with white thrombus (white arrow). (**d**,**e**) Proximal nonsignificant LAD lesion with SCP (white star) and healed plaque (blue arrow), calcified protrusion (blue arrowhead). (**f**) Proximal significant LM stenosis (AS = 84.4%) with eruptive CN and red thrombus (white arrowhead). (**C**) CA—final result after PCI with 3.0 mm DES/LM-LAD, 2.5 mm DES/diagonal branch.

**Figure 5:**
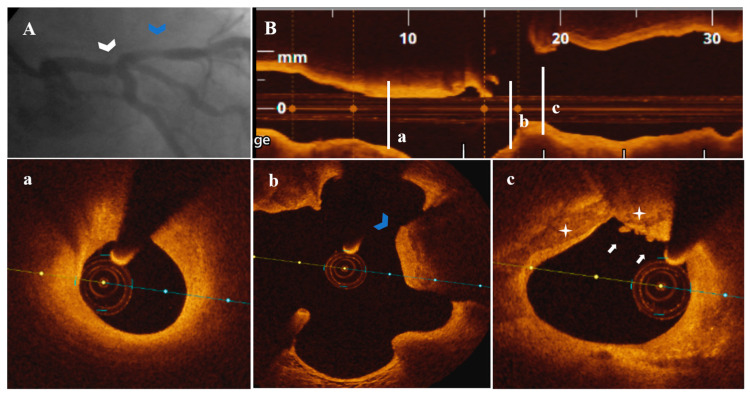
A 79-year-old patient presenting with UAP. (**A**) CA—Borderline distal LM (white arrowhead) and nonsignificant proximal LAD (blue arrowhead) stenoses. (**B**) OCT—LM-LAD plaque longitudinal view. (**a**) Nonsignificant LAD stenosis without marks of complication. (**b**) Calcified protrusion (blue arrowhead) following a (**c**) PE-SCP (white star) with white thrombi (white arrow) at the level of a nonsignificant LM stenosis (area stenosis = 44.9%).

**Figure 6:**
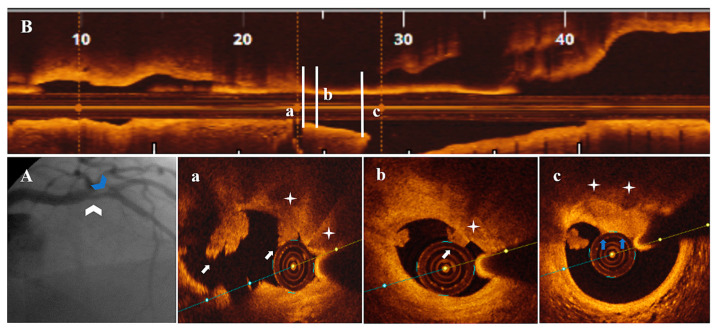
A 58-year-old patient presenting with NSTEMI. (**A**) CA—borderline proximal LAD stenosis (white arrowhead), severe first diagonal ostial stenosis (blue arrowhead). (**B**) OCT—LAD plaque longitudinal view. (**a**–**c**) Significant LAD lesion (area stenosis = 74.6%) presenting PE with white thrombi (white arrow) on SCP (white star) extending distally into the ostium of the first diagonal and proximally in interrelation with a healed plaque (blue arrow).

**Figure 7:**
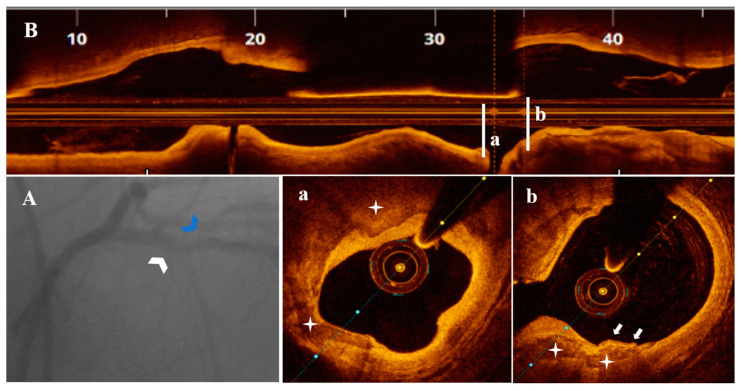
A 70-year-old patient presenting with UAP. (**A**) CA—borderline mid-LAD stenosis (white arrowhead), significant first diagonal ostial stenosis (blue arrowhead). (**B**) OCT—LAD plaque longitudinal view. (**a**,**b**) Nonsignificant LAD stenosis (area stenosis = 56.5%) presenting SCP (white star) with PE and red thrombus (white arrow).

Diagnostic CA findings are shown in Figure 1A, Figure 2A, Figure 3A, Figure 4A, Figure 5A, Figure 6A and Figure 7A. On CA, patients #1, #6 and #7 (Figure 1A, Figure 6A and Figure 7A) presented a borderline LAD stenosis, while patient #3 (Figure 3A) presented a borderline LM stenosis. Patient #2 (Figure 2A) presented a nonsignificant, eccentric and hazy ostial LAD plaque. In patient #5 (Figure 5A), there was a borderline LM stenosis and a nonsignificant and hazy LAD stenosis. Patient #4 (Figure 4A) demonstrated a significant LM stenosis and two sequential LAD stenoses, a nonsignificant and, respectively, a severe one. 

There was evidence of a grade 3 thrombolysis in myocardial infarction flow in all of the target vessels.

### 3.3. OCT Data

The OCT data of the seven patients are shown in Figure 1, Figure 2, Figure 3, Figure 4, Figure 5, Figure 6 and Figure 7. OCT confirmed lesion severity in patients #4 and #2, and in the rest of the cases which were borderline, reassessed the lesions as significant in patients #1 and #6. All of the culprit plaques were mainly calcific in nature (mean calcification length = 22.9 mm, 4-quadrant calcification in 3 cases). PE-SCP occurred mostly in the proximal plaque segment (71.4%) but not in the distal one and was associated with white thrombus in 85.7% of cases (6/7 patients).

PE-SCP presented with thrombus extending proximally in interrelation with an HCP aspect in 2/7 patients (Figure 3B,a,b and Figure 6B,a–c).

In 2/7 patients, PE-SCP coexisted with other acute marks of plaque disruption. As seen in Figure 4, there was a long LM-LAD plaque presenting PE-SCP with white thrombus (Figure 4c) and HCP (Figure 4d) in the significant and respectively nonsignificant LAD lesions, alongside eruptive CN (Figure 4a) and CP (Figure 4c,e). In the same patient, an eruptive CN with red thrombus determined the significant LM stenosis (Figure 4f). In Figure 5, there is PE-SCP with small thrombi (Figure 5c) followed by a CP (Figure 5b) at the LM level.

### 3.4. Treatment Strategy

PE-SCP lesions were treated conservatively in 4/7 patients, as nonsignificant lesions were identified on OCT. All 4 patients received potent dual antiplatelet therapy, high-dose statin, beta blockers, angiotensin-converting enzyme inhibitors/angiotensin receptor blockers with an uneventful in-hospital clinical evolution.

PCI with drug-eluting stent implantation was performed in patient #1 (Figure 1C) and #4 (Figure 4C) after adequate lesion preparation. Additional side-branch treatment with plain old balloon angioplasty in the former and two-stent technique in the latter was needed without any peri-procedural complications and with immediate symptomatic improvement. Patient #6 was referred to coronary artery bypass grafting with good postoperative evolution.

Long-term clinical follow-up was available in all patients. At a median follow-up period of 77 months, death occurred in four out of seven patients (two of which were treated medically).

## 4. Discussion

In this in vivo OCT study on the underlying mechanisms of ACS, PE-SCP lesions represent more than half of the total PE subset. The main clinical feature associated to PE-SCP is represented by NSTEMI or UAP. PE-SCP occur mostly on the LAD, are associated with nonsignificant OCT lesions, have generally a proximal intraplaque site, are predominantly complicated with white thrombi and are treated mainly medically in more than half of the cases.

PE is recognized as a distinct entity responsible for coronary thrombosis and is found in 23–31% of patients with ACS based on in vivo and OCT data [9,17]. When analyzing the underlying characteristics of eroded plaques, OCT studies have found a frequency of 50–56% for fibrous plaque and 44–50% for lipid plaque [9,10]. White and not red thrombus was identified in the majority (69%) of patients with PE [9]. In addition, Cao et al. [18] have shown that when comparing eroded to non-culprit plaques, calcium burden, when present, is higher in the former.

Alfonso et al. [19] were the first to report the case of an ACS patient with extensive superficial non-protruding calcification without signs of intimal rupture at the culprit lesion. PE with rather small red thrombi was identified at multiple sites.

Coronary calcification is typically regarded as a surrogate for advanced atherosclerosis with prognostic implications [20]. Although spotty calcification was found in the ruptured plaques of patients with ACS, suggesting a potential role in plaque vulnerability [21], the presence of more compact, dense calcium can reduce local shear stress and promote plaque stability [22]. Nonetheless, CN, a distinct manifestation of compact calcium is one of the accepted pathological substrates of ACS.

Expanding on the already known etiological paradigm of ACS, Sugiyama et al. [12] have further divided culprit calcified plaques into three subtypes: SCP, eruptive CN and CP. The SCP subtype was the most common feature among the three (67.4%), having a prevalence of 7.6% in the general ACS population, presenting as NSTEMI in 55% of cases. It was found mainly in the LAD, and it was associated with the smallest luminal diameter and the presence of white thrombus. What is more, post-PCI data show that the superficial calcium group had the smallest minimal stent area and highest rate of periprocedural myocardial infarction [23], emphasizing the need for intravascular imaging guided-PCI, more aggressive lesion preparation and also careful stent optimization.

On the other hand, at the 2-year follow-up lower rates of major adverse cardiovascular events, target-vessel myocardial infarction and ischemia-driven revascularization (10.1 vs. 13 vs. 32.1%, *p* = 0.001; 1.7 vs. 8.7 vs. 8.9%, *p* = 0.016; 5.6 vs. 13 vs. 16.1%, *p* = 0.029) have been reported when comparing SCP to CP and eruptive CN [24]. At multivariate analysis, eruptive CN was a very strong predictor of major adverse cardiovascular events (HR: 3.14; 95% CI: 1.64–6.02; *p* = 0.001) [12].

In our study, PE-SCP showed a strong association with white thrombus (85.7%) that had mostly a proximal (71.4%) intraplaque topography. Yamamoto et al. [25] have demonstrated using a fluid dynamic model integrating the OCT data of PE patients that high endothelial shear stress promotes thrombus formation, which then propagates distally to regions of high oscillatory shear index. A possible explanation for the etiopathology of these cases could reside in the increased structural stress at the junction of the rigid calcified plate with a thin fibrous cap causing intimal microdisruptions, thus generating thrombosis [26].

Of particular interest, as seen in patient #4, several complication sites can be found in the same patient and even in the same artery, thus making the culprit troublesome to identify. Furthermore, in patients #3, #4 and #6, the culprit coexisted with an HCP aspect. This concept is easy to understand, as HCP is the hallmark of previous plaque destabilization. HCP had an overall incidence of 40% in a population of ACS and stable angina pectoris patients, as shown in a recent metanalysis of 13 studies emphasizing the dynamic and often silent process of plaque disruption–stabilization constantly taking place in the coronary system [27].

A conservative medical treatment was employed in more than half of the cases due to nonsignificant lesions evaluated by OCT with complete symptom resolution and good in-hospital clinical evolution. As shown in the EROSION study, over 90% of patients treated with aspirin and ticagrelor remain free of major adverse cardiovascular events at 1 year, while complete thrombus resolution is noted in almost half of the cases [28].

It is worth mentioning that all of our PE-SCP patients were on prior chronic single antiplatelet and statin therapy, which could have provided a “protective” effect against plaque rupture. Furthermore, impaired imaging conditions in the context of a large and occlusive thrombus could have determined the operator to limit the use of OCT. As both plaque rupture and a large thrombotic mass are seen mainly in STEMI patients [29], it could provide a possible explanation as to why no STEMI was found in our patient cohort.

Several limitations of this study are worth mentioning. First, this is an observational retrospective study with a small patient cohort; therefore, conclusions can only be considered exploratory. Second, imaging follow-up was not available; thus, the long-term significance of our study is yet to be known. Third, a functional evaluation of lesion significance was not performed, and it would have been of interest. Fourth, OCT discrimination between CN and red thrombus is sometimes cumbersome due to high attenuation, in which case adjacent frames were analyzed. The detection of additional dense calcium would make the diagnosis of CN more likely. Further large-scale studies are needed to fully clarify PE-SCP pathophysiological mechanisms and long-term outcomes.

## 5. Conclusions

SCP are associated to more than half of the PE culprit lesions in ACS patients. SCP are to be considered as calcium-related risk plaques, adding to the known paradigm correlating plaque vulnerability only to spotty calcification and CN. PE-SCP occur mostly on the LAD, correlate with white thrombi and have mainly a proximal plaque site. In more than half of the cases, PE-SCP relate to nonsignificant OCT lesions and lead to conservative treatment.

## Figures and Tables

**Table 1:** Baseline patient characteristics.

Variable	Patient 1	Patient 2	Patient 3	Patient 4	Patient 5	Patient 6	Patient 7
Age, years	74	69	51	79	79	58	70
Gender	F	F	M	F	M	F	M
Diagnosis	UAP	UAP	NSTEMI	NSTEMI	UAP	NSTEMI	UAP
Risk factors							
Hypertension	Yes	Yes	Yes	Yes	Yes	Yes	Yes
Diabetes mellitus	No	No	No	Yes	No	Yes	Yes
Hyperlipidemia	Yes	Yes	Yes	Yes	No	Yes	No
Smoking habit	No	No	Yes	No	No	Yes	No
Overweight	No	Yes	No	No	No	No	No
Clinical history							
Previous MI	No	No	Inferior MI	No	Inferior MI	Inferior MI	No
Previous PCI	No	No	BMS/Cx	DES/RCA	BMS/RCA	DES/LM; BMS/RCA	No
Previous stroke	No	No	No	Yes	Yes	Yes	No
PAD	No	No	Yes	No	Yes	No	Yes
CKD	No	No	No	Yes	Yes	No	No
Atrial fibrillation	Yes	No	No	Yes	No	No	No
Clinical presentation at admission	Daily resting angina the past 2 weeks	De-novo resting angina and dyspnea	Ongoing prolonged angina and ventricular tachycardia	24 h after diagnosis of high-risk NSTEMI	Recurrent angina episodes at rest the past 3 days	Prolonged resting angina; exercise angina for 1 year	Worsening angina the last 2 weeks
Physical examination	Grade II systolic murmur RUSB	Normal	Absent pulses left femoral artery	Normal	Absent pulses left popliteal artery	Normal	Low-grade systolic murmur RUSB; diminished bilateral distal pulses
Previous medication							
Aspirin	Yes	Yes	Yes	Yes	Yes	Yes	Yes
Statin	Yes	Yes	Yes	Yes	Yes	Yes	Yes
Medication at discharge							
Aspirin	Yes	Yes	Yes	Yes	Yes	Yes	Yes
P2Y_12_ inhibitor	Yes	Yes	Yes	Yes	Yes	No	Yes
Statin	Yes	Yes	Yes	Yes	Yes	Yes	Yes
Beta blocker	Yes	Yes	Yes	Yes	Yes	Yes	Yes
ACEI/ARB	Yes	Yes	Yes	No	Yes	Yes	Yes
Electrocardiography	Right bundle branch block + left anterior hemiblock	Voltage criteria left ventricular hypertrophy	Normal	ST segment depression V4-V6, DI, aVL	Right bundle branch block	Normal	Negative T waves V4-V6, DI, aVL
Echocardiogram	Moderate aortic stenosis, EF 55%	Normal, EF 60%	Left ventricular dilation, inferior akinesia, anterolateral hypokinesia, EF 30–35%	Basal septum and inferolateral hypokinesia, EF 40%	Inferior wall hypokinesia, EF 45%	Septum and anterolateral kypokinesia, EF 45%	Inferior wall akinesia, mild aortic stenosis, EF 45%
Laboratory data							
TC, mg/dL	232	215	149	154	152	182	94
LDL-C, mg/dL	157	142	75	102	80	109	18
HDL-C, mg/dL	48	58	33	39	41	45	20
Triglycerides, mg/dL	136	409	206	216	157	140	279
Creatinine, mg/dL	1.03	0.9	0.77	1.97	1.22	0.7	0.88
Peak CK-MB, U/L	11	8	77	127	19	101	24
Leucocytes, 10^9^/L	8.7	12.6	6.89	10.4	6.25	7.09	8.46
Hemoglobin, g/dL	14	14.3	14.2	12.3	12	14.4	13.3

ACEI: angiotensin-converting enzyme inhibitor. ARB: angiotensin receptor blocker. BMS: bare-metal stent. CKD: chronic kidney disease. CK-MB: creatin-kinase MB isoform. Cx: circumflex artery. DES: drug-eluting stent. EF: ejection fraction. HDL-C: high-density lipoprotein cholesterol. LDL-C: low-density lipoprotein cholesterol. LM: left main coronary artery. MI: myocardial infarction. NSTEMI: non-ST-segment elevation myocardial infarction. PAD: peripheral arterial disease. PCI: percutaneous coronary intervention. RCA: right coronary artery. RUSB: right upper sternal border. TC: total cholesterol. UAP: unstable angina pectoris.

**Table 2:** Imaging findings.

Variable	Patient 1	Patient 2	Patient 3	Patient 4	Patient 5	Patient 6	Patient 7
Angiographic data							
Culprit vessel	LAD	LAD	LM	LM-LAD	LM	LAD	LAD
Multivessel disease *	Yes; LAD and D2	No	No	Yes; LM, LAD, D2, CTO Cx (patent RCA stent)	Yes; D1, OM1 (patent RCA stent)	Yes; LAD, D1 (patent LM and RCA stents)	Yes; D1 and CTO RCA
Lesion length, mm	32	23	25	45	27	28	40
Lesion severity, %	50	30	40	55 (LM)/80 (mid-LAD)	40	50	50
TIMI flow	3	3	3	3	3	3	3
OCT data							
Reference lumen diameter, mm	2.53	3.08	4.66	4.1 (LM)/2.99 (LAD)	3.91	2.38	3.55
MLD, mm	0.66	1.72	2.85	1.22 (LM)/0.58 (mid-LAD)	2.13	0.70	1.86
Diameter stenosis, %	74	44.1	38.8	70.2 (LM)/80.6 (mid-LAD)	45.5	70.6	47.6
Reference lumen area, mm^2^	5.02	7.44	17.14	13.2 (LM)/7.01 (LAD)	12.8	4.45	9.9
MLA, mm^2^	1.31	4.61	9.77	2.06 (LM)/1.22 (mid-LAD)	7.05	1.13	4.3
Area stenosis, %	73.9	38	42.9	84.4 (LM)/82.6 (mid-LAD)	44.9	74.6	56.5
Calcification length, mm	28	10	25	39	12	14	32
Calcification quadrant, n	4	2	3	4	2	2	4
Thrombus type	White	White	White	White/red	White	White	Red
Thrombus intraplaque site	Proximal/medial	Proximal	Proximal	Medial	Proximal	Proximal	Medial
Procedural data							
Number of stents, n	1	N/A	N/A	1	N/A	N/A	N/A
Stent length, mm	23	N/A	N/A	40	N/A	N/A	N/A
Stent diameter, mm	2.75	N/A	N/A	3	N/A	N/A	N/A
Management	PCI	Medical	Medical	PCI	Medical	coronary artery bypass grafting	Medical

CTO: chronic total occlusion. Cx: circumflex coronary artery. D: diagonal branch. LAD: left anterior descending coronary artery. LM: left main coronary artery. MLA: minimum lumen area. MLD: minimum lumen diameter. OCT: optical coherence tomography. PCI: percutaneous coronary intervention. RCA: right coronary artery. TIMI: thrombolysis in myocardial infarction. * Significant (>70% quantitative coronary angiography) stenosis in any of the non-culprit vessels or LM disease.

## Data Availability

The data presented in this study are available on request from the corresponding author. The data are not publicly available because they are property of Cluj County Emergency Hospital, Cluj-Napoca, Romania.
